# ERK Signaling Is Essential for Macrophage Development

**DOI:** 10.1371/journal.pone.0140064

**Published:** 2015-10-07

**Authors:** Edward T. Richardson, Supriya Shukla, Nancy Nagy, W. Henry Boom, Rose C. Beck, Lan Zhou, Gary E. Landreth, Clifford V. Harding

**Affiliations:** 1 Department of Pathology, Case Western Reserve University and University Hospitals Case Medical Center, Cleveland, Ohio, United States of America; 2 Medical Scientist Training Program, Case Western Reserve University, Cleveland, Ohio, United States of America; 3 Division of Infectious Diseases and HIV Medicine, Case Western Reserve University and University Hospitals Case Medical Center, Cleveland, Ohio, United States of America; 4 Center for AIDS Research, Case Western Reserve University and University Hospitals Case Medical Center, Cleveland, Ohio, United States of America; 5 Department of Neurosciences, Case Western Reserve University, Cleveland, Ohio, United States of America; Emory University, UNITED STATES

## Abstract

Macrophages depend on colony stimulating factor 1 (also known as M-CSF) for their growth and differentiation, but the requirements for intracellular signals that lead to macrophage differentiation and function remain unclear. M-CSF is known to activate ERK1 and ERK2, but the importance of this signaling pathway in macrophage development is unknown. In these studies, we characterized a novel model of *Erk1* ^*-/-*^ *Erk2* ^*flox/flox*^ *Lyz2* ^*Cre/Cre*^ mice in which the ERK2 isoform is deleted from macrophages in the background of global ERK1 deficiency. Cultures of M-CSF-stimulated bone marrow precursors from these mice yielded reduced numbers of macrophages. Whereas macrophages developing from M-CSF-stimulated bone marrow of *Erk2* ^*flox/flox*^ *Lyz2* ^*Cre/Cre*^ mice showed essentially complete loss of ERK2 expression, the reduced number of macrophages that develop from *Erk1* ^*-/-*^ *Erk2* ^*flox/flox*^ *Lyz2* ^*Cre/Cre*^ bone marrow show retention of ERK2 expression, indicating selective outgrowth of a small proportion of precursors in which Cre-mediated deletion failed to occur. The bone marrow of *Erk1* ^*-/-*^ *Erk2* ^*flox/flox*^ *Lyz2* ^*Cre/Cre*^ mice was enriched for CD11b^+^ myeloid cells, CD11b^hi^ Gr-1^hi^ neutrophils, Lin^-^ c-Kit^+^ Sca–1^+^ hematopoietic stem cells, and Lin^-^ c-Kit^+^ CD34^+^ CD16/32^+^ granulocyte-macrophage progenitors. Culture of bone marrow Lin^-^ cells under myeloid-stimulating conditions yielded reduced numbers of monocytes. Collectively, these data indicate that the defect in production of macrophages is not due to a reduced number of progenitors, but rather due to reduced ability of progenitors to proliferate and produce macrophages in response to M-CSF-triggered ERK signaling. Macrophages from *Erk1* ^*-/-*^ *Erk2* ^*flox/flox*^ *Lyz2* ^*Cre/Cre*^ bone marrow showed reduced induction of M-CSF-regulated genes that depend on the ERK pathway for their expression. These data demonstrate that ERK1/ERK2 play a critical role in driving M-CSF-dependent proliferation of bone marrow progenitors for production of macrophages.

## Introduction

Macrophages are important phagocytes that internalize and degrade extracellular debris and pathogens, express pro-inflammatory and regulatory cytokines and present antigens via MHC molecules for recognition by T lymphocytes. Macrophages that reside in different tissues are known by different names, such as microglia (brain), Kupffer cells (liver), Langerhans cells (skin), or macrophages (many sites) [1]. Macrophages in different tissues arise from precursors at different embryonic or adult stages. For example, microglia arise from yolk sac precursors, while blood monocytes and intestinal macrophages arise from differentiation of adult hematopoietic stem cells in the bone marrow [2]. Circulating monocytes in the adult animal differentiate into macrophages upon emigration into tissues; this process is a component of acute inflammation and is required for tissue remodeling and healing.

The development of macrophages depends on the cytokine M-CSF (also known as CSF1) and the M-CSF receptor (also known as CSF1R or CD115). Mice deficient in M-CSF, originally characterized as a spontaneous mutation *Csf1* ^*op/op*^ [3], lack monocytes, macrophages, and osteoclasts, which are specialized macrophage-derived phagocytes involved in bone remodeling. These studies and subsequent research on mice lacking M-CSF receptor [4] have confirmed the important and essential role of M-CSF in macrophage development. The M-CSF receptor homodimerizes following ligation of M-CSF to form an active tyrosine kinase, which then triggers intracellular signaling cascades, including the PI3K/Akt [5] and ERK and JNK MAP kinase pathways [6–9]. Despite the fact that M-CSF triggers several broad pathways involved in growth factor responses and cell survival, the requirements for any individual pathway are not known. Here, we sought to explore the specific role of the ERK pathway in macrophage development and proliferation.

ERK1 and ERK2 are serine/threonine kinases involved in numerous cellular pathways, including growth and development, and ERK phosphorylation is triggered by activation of receptor tyrosine kinases in response to growth factor signals. M-CSF receptor signaling leads to ERK activation, as well as activation of other signaling pathways [10]. In studies with cell lines stimulated with M-CSF, ERK was found to transmit signals leading to macrophage commitment from the M-CSF receptor [8], but these studies were limited to cell lines, and the role of ERK in macrophage growth and differentiation from bone marrow progenitors is unexplored.

For these studies, we attempted to create mice in which macrophages would lack both ERK1 and ERK2 activity. While *Erk1* ^*-/-*^ mice are viable, *Erk2* ^*-/-*^ mice do not survive [11], so we developed macrophage lineage-directed knockout of the ERK2 isoform in the background of a germline ERK1 knockout mouse. We used LysM-driven Cre recombinase to target deletion in mature myeloid cells, as the LysM promoter has been characterized as active only in mature monocytes, macrophages, and neutrophils, and not in immature monocytic lineage cells, lymphoid or erythroid lineages, or immature hematopoietic stem cells [12]. Additionally, LysM-Cre expression in tissues was determined by Jackson Laboratories and is myeloid-specific. We crossed *Erk2* ^*flox/flox*^ mice to *Lyz2* ^*Cre/Cre*^ mice, which express Cre recombinase in myeloid progenitors and macrophages [13], and observed efficient deletion of *Erk2* in macrophages derived from M-CSF-stimulated bone marrow. Therefore, macrophages from *Erk1* ^-/-^ *Erk2* ^*flox/flox*^ *Lyz2* ^*Cre/Cre*^ bone marrow were expected to show deletion of both ERK1 and ERK2 isoforms in myeloid lineage cells, especially macrophages. Surprisingly, these macrophages expressed ERK2, and were able to respond to TLR2 signaling with induction of ERK-regulated genes, e.g. *Il10* and *Il12b*, much like wild-type macrophages. These results imply a selective outgrowth of macrophages from a minor population of progenitors in which deletion of floxed *Erk2* did not occur, which demonstrates the necessity of the ERK pathway for the development of macrophages from M-CSF-stimulated progenitors. We conclude that the M-CSF receptor pathway requires signaling through ERK to drive macrophage development.

## Materials and Methods

### Ethics Statement

The Institutional Animal Care and Use Committee of Case Western Reserve University reviewed and approved all experiments (protocol number: 2012–0007). Mice were housed in specific-pathogen-free conditions in groups of five animals; they were provided a 12-hour light-dark cycle, bedding and nesting materials, and food and water *ad libitum*. Mice were monitored for health and distress at least twice weekly, and remained untouched until sacrifice (by CO_2_ inhalation) for *ex vivo* cell collections; no early humane endpoints were needed. All efforts were employed to maintain animal welfare.

### Mouse strains and cell culture methods

Wild-type C57BL/6J mice and LysM-Cre knock-in mice on a C57BL/6 background (*Lyz2* ^*Cre/Cre*^ [13], herein referred to as LysM) were obtained from Jackson Laboratories (Bar Harbor, ME). *Erk1* ^*-/-*^ and *Erk2* ^*flox/flox*^ mutant mice on a C57BL/6 background were generated as described previously [14, 15]. First, single mutant *Erk1* ^*-/-*^ and *Erk2* ^*flox/flox*^ mice were each crossed with *Lyz2* ^*Cre/Cre*^ mice to generate double mutant *Erk1* ^*-/-*^ *Lyz2* ^*Cre/Cre*^ mice (used further as breeding stock) and *Erk2* ^*flox/flox*^ *Lyz2* ^*Cre/Cre*^ (used as breeding stock, and as an experimental ERK2 single knockout in the myeloid lineage to complement the *Erk1* ^*-/-*^ single knockout). Second, these double mutant mice were crossed with each other to produce F1 *Erk1* ^*+/-*^ *Erk2* ^*+/flox*^ *Lyz2* ^*Cre/Cre*^ mice. Sibling crosses of these F1 mice produced *Erk1* ^*-/-*^ *Erk2* ^*flox/flox*^ *Lyz2* ^*Cre/Cre*^ mice (herein referred to as ERK1/2) in Mendelian ratios, and these were selected and bred. Genotypes were confirmed by PCR as described below. Mice were used for experiments at 12–16 weeks of age and were sex-matched within a single experiment.

Bone marrow cells were harvested from femurs and tibias and red blood cells were lysed in ACK lysing buffer (Lonza, Walkersville, MD). Bone marrow-derived macrophages were derived from bone marrow cells that were cultured for 7 d in DMEM (HyClone, Logan, UT) supplemented with 10% heat-inactivated fetal bovine serum (Gibco, Carlsbad, CA), 50 μM 2-ME (Bio-Rad, Hercules, CA), 1 mM sodium pyruvate (HyClone), 10 mM HEPES (HyClone), 100 units/ml penicillin, 100 μg/ml streptomycin (HyClone) (complete medium, referred to as D10F), and 25% conditioned medium from the LADMAC cell line, a source of M-CSF [16]. Bone marrow cell suspensions and bone marrow macrophage cell numbers were counted using Trypan Blue exclusion (Sigma, St. Louis, MO) and a hemacytometer.

Bone marrow macrophages were cultured in D10F for experiments. Where indicated, macrophages were treated with cytokines or compounds at doses recommended by the manufacturer. These included recombinant murine M-CSF (2 ng/ml final concentration; R&D Systems, Minneapolis, MN), synthetic lipopeptide Pam_3_CSK_4_ (30 nM final concentration; Invivogen, San Diego, CA), the MEK inhibitor U0126 (10 μM final concentration; Calbiochem, Billerica, MA), or an equivalent volume of DMSO (Sigma) as a vehicle control for U0126.

To assess the potential for myeloid progenitor differentiation under *in vitro* conditions, we performed magnetic bead labeling and depletion of bone marrow lineage marker-positive cells (Lineage Cell Depletion Kit, Miltenyi Biotec, Auburn, CA), and 2.5–10 × 10^4^ Lin^-^ cells (containing hematopoietic stem cells and myeloid progenitors) were grown in 24-well plates in IMDM (Gibco) supplemented with 10% heat-inactivated fetal bovine serum (Gibco), 100 units/ml penicillin, and 100 μg/ml streptomycin (HyClone) with the addition of recombinant murine stem cell factor (SCF, R&D Systems, 10 ng/ml), recombinant murine IL–3 (R&D Systems, 5 ng/ml), recombinant murine IL–6 (R&D Systems, 10 ng/ml), and recombinant murine M-CSF (R&D Systems, 5 ng/ml). Cells were analyzed daily by counting using Trypan Blue exclusion, by flow cytometry for monocyte (CD11b^+^/Gr–1^-^) and granulocyte (CD11b^+^/Gr–1^+^) differentiation markers, and by morphologic evaluation of cytospin preparations (stained with a Hema 3 kit; Fisher Scientific, Pittsburgh, PA).

### Genotyping

Mouse tail tissue was subjected to DNA extraction using the REDExtract-N-Amp Tissue PCR Kit (Sigma Aldrich, St. Louis, MO), and PCR was performed using HotStarTaq Plus Master Mix Kit (Qiagen, Valencia, CA). Primers for genotyping the *Erk1* locus were: Ex6F (5’-GTA TCT TGG GTT CCC CAT CC–3’), Neo7F (5’-GGG GAA CTT CCT GAC TAG GG–3’), and Ex8R (5’-GCT CCA TGT CGA AGG TGA AT–3’). Primers for the *Erk2* locus were: E2F-U (5’-AGC CAA CAA TCC CAA ACC TG–3’) and E2F-L (5’-GGC TGC AAC CAT CTC ACA AT–3’). Primers for the *Lyz2* locus were: oIMR3066 (5’-CCC AGA AAT GCC AGA TTA CG–3’) and oIMR3067 (5’-CTT GGG CTG CCA GAA TTT CTC–3’). The *Erk1* PCR cycling conditions were: 94°C for 4 min, and then 40 cycles of 94°C for 30 s, 60°C for 30 s, and 72°C for 30 s, followed by a final extension step at 72°C for 10 min. The *Erk2* PCR conditions were: 94°C for 4 min, and then 35 cycles of 94°C for 15 s, 52.5°C for 15 s, and 72°C for 30 s, followed by a final extension step at 72°C for 10 min. The *Lyz2* PCR conditions were: 94°C for 3 min, and then 35 cycles of 94°C for 30 s, 62°C for 1 min, and 72°C for 1 min, followed by a final extension step at 72°C for 2 min. The *Erk1* and *Erk2* PCR reactions were performed on a C1000 instrument (Bio-Rad, Hercules, CA), and the *Lyz2* PCR was performed on a Mastercycler instrument (Eppendorf, Hamburg, Germany). PCR products were run on 1% agarose gels in Tris-acetate-EDTA buffer (Invitrogen, Carlsbad, CA), stained with ethidium bromide (Fisher Scientific), and imaged with a Gel Doc instrument (Bio-Rad).

### Western blotting

Macrophages (10^6^ per condition) were treated as indicated, washed once with ice cold PBS, and lysed with RIPA buffer (50 mM Tris pH 7.5, 150 mM NaCl, 1% NP–40, 0.5% sodium deoxycholate, 0.1% SDS) with addition of a protease and protein phosphatase inhibitor cocktail (Pierce, Rockford, IL). Lineage-depleted bone marrow cells and FACS-isolated bone marrow monocytes and granulocytes were lysed as above. Samples were boiled in reducing sample buffer (Bio-Rad) and run on SDS-PAGE gels (Bio-Rad), transferred to polyvinylidene difluoride membranes (Millipore, Billerica, MA), and blocked in 5% nonfat dry milk in PBS–0.1% Tween 20. Primary antibodies included anti-phospho-ERK1/2 (clone D13.14.4E), anti-ERK1/2 (clone 137F5), and anti-β-actin (clone D6A8) (all from Cell Signaling Technology, Danvers, MA). Primary antibodies were incubated in blocking buffer overnight at 4°C; membranes were then washed three times in PBS–0.1% Tween 20, treated with secondary antibody (horseradish peroxidase-linked goat anti-rabbit IgG, catalog number 7074, Cell Signaling Technology) in blocking buffer, washed three times, and treated with enhanced chemiluminescence reagent (Pierce). Membranes were exposed to film (Amersham GE Healthcare, Pittsburgh, PA), and images were scanned and analyzed. Densitometric analysis was performed using ImageJ software [17].

### Complete blood counts

Blood was collected from the tail veins of mice by nicking the tails with a razor blade and collecting the blood dropwise directly into MAP capillary blood collection tubes with K2EDTA anticoagulant (BD, Franklin Lakes, NJ). Blood counts were analyzed on a Hemavet 950FS instrument (Drew Scientific, Waterbury, CT).

### Flow cytometry and Fluorescence-Activated Cell Sorting (FACS)

Flow cytometry was performed on bone marrow-derived macrophages, *ex vivo* bone marrow preparations, and myeloid cells derived by *in vitro* culture of Lin^-^ bone marrow cells. For experiments with bone marrow-derived macrophages, cells were first incubated with 2 μg/ml Fc Block in PBS with 1% BSA for 15 min on ice; *ex vivo* bone marrow experiments were performed without this step, as CD16/32 was a marker of interest. Cells were then incubated with conjugated primary antibodies in PBS–1% BSA at 2 μg/ml final concentration for 30 min on ice (extended to 90 min on ice when detecting CD34). Cells were washed in PBS without BSA three times, and then chemically labeled with LIVE/DEAD Fixable Yellow cell viability reagent according to the manufacturer’s instructions (Invitrogen, Carlsbad, CA). Cells were then washed twice more in PBS, fixed in 1% formaldehyde (Pierce), washed once more and resuspended in PBS–1% BSA, and analyzed on an LSR II flow cytometer (BD Biosciences, San Jose, CA) or an Accuri C6 flow cytometer (BD Biosciences). Bone marrow-derived macrophages and differentiated myeloid cells from bone marrow cultures were not stained with LIVE/DEAD reagent; viability of cells was instead verified prior to use in an experiment using Trypan Blue exclusion.

Automated compensation was performed at the time of data acquisition on the LSR II instrument using appropriate singly stained controls. CD16/32 and LIVE/DEAD controls were generated using *ex vivo* bone marrow cells and the above staining protocols. All others were generated using anti-rat/hamster Ig, κ CompBeads (BD Biosciences) according to the manufacturer’s instructions. Gating cut-offs were set using fluorescence minus one controls, and the gating strategy was adopted from Mayle et al [18] and Challen et al [19]. Analysis and post-acquisition compensation for data generated on the Accuri C6 instrument were done in FlowJo version X (Tree Star, Ashland, OR).

For FACS isolation of bone marrow monocytes and neutrophils, bone marrow cells were subjected to red blood cell lysis, treated with Fc block for 15 min on ice, and labeled with 2 μg/ml TER–119, B220, CD11b and Gr–1 for 30 min on ice. Cells were washed twice and resuspended in PBS–1% BSA. FACS was performed with a FACSAria II instrument (BD Biosciences). TER–119^+^ and B220^+^ cells were excluded; CD11b^+^/Gr–1^-^ monocytes and CD11b^hi^/Gr-1^hi^ neutrophils were isolated for Western blot analysis.

### Quantitative reverse transcriptase PCR (qRT-PCR)

Macrophages (10^6^ per condition) were incubated with U0126, M-CSF, or Pam_3_CSK_4_ and washed once in cold PBS. RNA was extracted using the RNeasy Plus Mini Kit (Qiagen). RNA concentrations were determined by absorbance at 260 nm using a NanoDrop 2000 (Thermo Scientific, Wilmington, DE). Total RNA was reverse transcribed using the QuantiTect Reverse Transcription kit (Qiagen), and 200 ng of total cDNA was then amplified by real-time PCR using iQ SYBR Green Supermix (Bio-Rad) on a CFX96 instrument (Bio-Rad). Primers were for *Gapdh* (forward: 5’-AAC GAC CCC TTC ATT GAC–3’, reverse: 5’-TCC ACG ACA TAC TCA GCA C–3’ [20]) *Il10* (forward: 5’-AGA GAA GCA TGG CCC AGA AAT C–3’, reverse: 5’- TCA TGG CCT TGT AGA CAC CTT G–3’ [21]), *Il12b* (forward: 5’-AGA AAG GTG CGT TCC TCG TAG–3’, reverse: 5’-AGC CAA CCA AGC AGA AGA CAG–3’ [21]), *Cd33* (forward: 5’-GAC CAT CCA GCT CAA TGT TAC CC–3’, reverse: 5’-AGA CAG AGC CCA AGA ATC AGG A–3’ [22]), and *Dusp5* (forward: 5’-GAA GTG CCT ACC ACG CAT CC–3’, reverse: 5’-TCC GGC GGG AAA CAT TCA G–3’, PrimerBank ID 145966802c2 [23]). Real-time PCR conditions included a hot start at 95°C for 3 min, followed by 50 cycles of 95°C for 10 s, 59°C for 10 s, and 72°C for 30 s, and a post-amplification melting curve from 65°C to 95°C in steps of 0.5°C per 5 s. Transcript quantities were determined relative to *Gapdh* expression with the formula 2^-[Ct(target gene)-Ct(Gapdh)]^ [24].

### Statistical analysis

Statistics were calculated using GraphPad Prism 5. Statistical significance was determined using Student’s *t* test.

## Results

### ERK expression is critical for development of macrophages in M-CSF-stimulated bone marrow cultures

**Fig 1:**
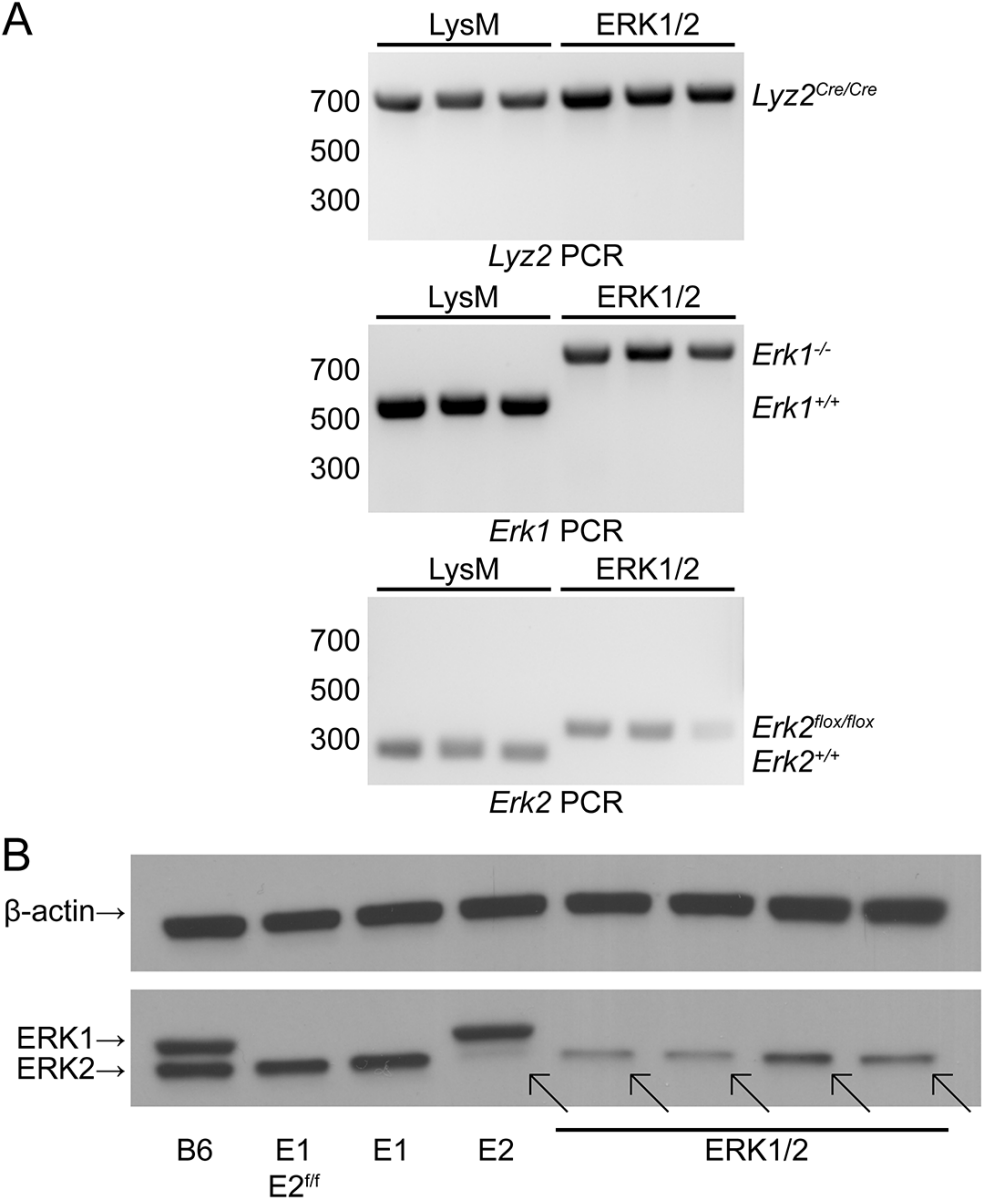
LysM and ERK1/2 mice express the expected mutations in germline DNA, but macrophages from ERK1/2 mice selectively retain expression of ERK2 protein. (A) DNA from clipped tails of LysM and ERK1/2 mice (three of each) was amplified to detect the mutant or wild-type alleles for *Lyz2*, *Erk1*, and *Erk2*. (B) Macrophages were derived from bone marrow cultures stimulated for 7 d with LADMAC-conditioned medium containing M-CSF, and expression of ERK1 and ERK2 was detected by Western blotting. Diagonal arrows indicate the position of the ERK2 band, expressed in ERK1/2 macrophages but greatly reduced in *Erk2* ^*flox/flox*^ *Lyz2* ^*Cre/Cre*^ macrophages. Results are representative of three independent experiments. Labels: B6, C57BL/6J; E1 E2^f/f^, *Erk1* ^*-/-*^ *Erk2* ^*flox/flox*^; E1, *Erk1* ^*-/-*^; E2, *Erk2* ^*flox/flox*^ *Lyz2* ^*Cre/Cre*^.

We began these studies by producing mice harboring three homozygous mutations, *Erk1* ^*-/-*^ *Erk2* ^*flox/flox*^ *Lyz2* ^*Cre/Cre*^ (referred to as ERK1/2 mice). Deletion of floxed *Erk2* by LysM-driven expression of Cre recombinase was predicted to produce myeloid lineage cells (macrophages, neutrophils, and myeloid dendritic cells) deficient in both ERK1 and ERK2. Accordingly, we tested ERK1 and ERK2 expression at the protein level in macrophages from these triple mutant mice and mice with only one or two of these genetic alterations. Genetic analysis of tail tissue from LysM (*Lyz2* ^*Cre/Cre*^) and ERK1/2 mice confirmed that the mice had the expected mutations (Fig 1A). Both LysM and ERK1/2 mice showed the 700 bp band indicating *Lyz2* ^*Cre/Cre*^ homozygosity. LysM mice expressed the wild-type 526 bp band for *Erk1*, while ERK1/2 mice with the *Erk1* knockout insert showed the predicted band at 749 bp. LysM mice showed the wild-type 275 bp band for *Erk2*, and ERK1/2 mice showed the predicted *Erk2* mutant band at 350 bp (Fig 1A). When we studied macrophages from bone marrow stimulated with M-CSF-containing LADMAC conditioned medium, Western blots showed that macrophages from ERK1/2 mice retained ERK2 protein expression at approximately 25% of the level seen in wild-type or *Erk1* single knockout macrophages (by densitometry; Fig 1B shows macrophages from four individual ERK1/2 mice from four distinct breeder pairs). Notably, *Erk2* ^*flox/flox*^ *Lyz2* ^*Cre/Cre*^ macrophages (labeled “E2”, fourth lane in Fig 1B) showed very near complete absence of ERK2 protein expression, indicating that Cre expression driven by the LysM promoter in macrophages could efficiently mediate deletion of floxed *Erk2*. This demonstrates that macrophages can develop in the absence of ERK2 when ERK1 expression is retained. Together, these data indicate that *Lyz2* ^*Cre/Cre*^ provides efficient deletion of floxed ERK2 in macrophages when ERK1 is present, but expression of ERK2 is retained in absence of ERK1, implying selective outgrowth of a minor progenitor population in which *Erk2* deletion was incomplete. The decreased expression levels of ERK2 seen in these cells may be due to deletion of one copy of floxed *Erk2* in these cells or other mechanisms. We conclude that ERK activity is critical for development of macrophages in M-CSF-stimulated bone marrow cultures. This activity can be provided by either ERK1 or ERK2; expression of one or both of these proteins is required for normal macrophage development.

### ERK activity is required for induction of some genes triggered by M-CSF

**Fig 2:**
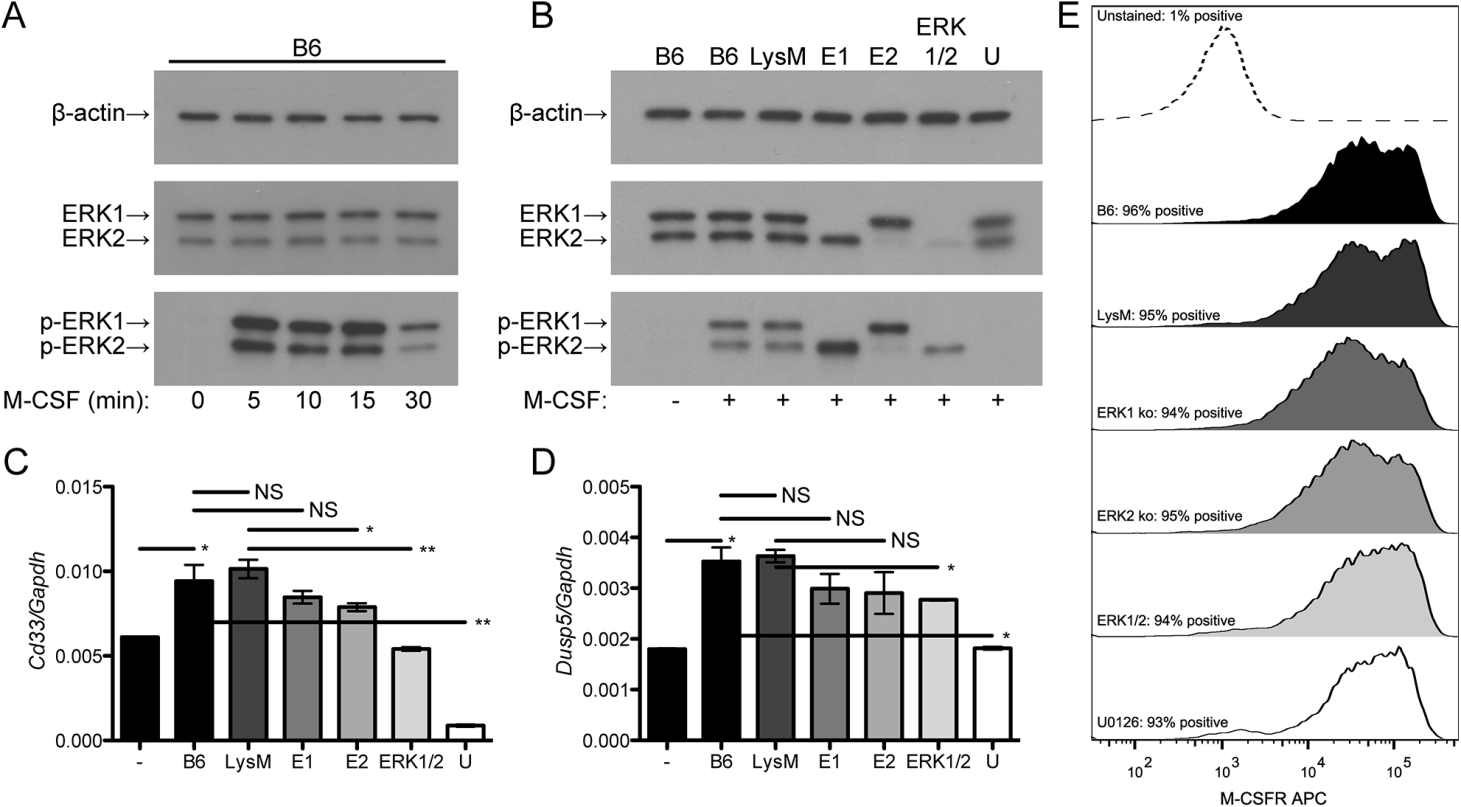
ERK signaling is required for some macrophage responses to M-CSF. (A) Wild-type bone marrow-derived macrophages were treated with 2 ng/ml M-CSF for the indicated times, lysed, and analyzed by Western blotting. Data represent two independent experiments. (B) Bone marrow-derived macrophages were pre-treated for 30 min with U0126 or DMSO vehicle (all others) and then treated with 2 ng/ml M-CSF for 10 min, lysed, and analyzed by Western blotting. Densitometric values (in arbitrary units) for LysM, E1 and ERK1/2 respectively were 33.7, 31.9 and 32.3 for β-actin; 43.7, 36.1 and 6.81 for ERK2; and 31.3, 54.9 and 14.0 for p-ERK2. Data represent two independent experiments. (C) and (D) Bone marrow-derived macrophages were pre-treated for 30 min with U0126 or DMSO and then treated with 2 ng/ml M-CSF for 24 h. RNA expression was measured by qRT-PCR. Each data point represents mean ± standard deviation of triplicate samples. Data are representative of three independent experiments. (E) Bone marrow macrophages were pre-treated with U0126 or DMSO, treated with 2 ng/ml M-CSF for 24 h, collected by detaching the cells using PBS with 10 mM EDTA, stained with 2 μg/ml anti-CD115, and analyzed by flow cytometry. Data represent two independent experiments. Labels: -, untreated; +, treated with M-CSF; B6, C57BL/6J; E1, *Erk1* ^*-/-*^; E2, *Erk2* ^*flox/flox*^ *Lyz2* ^*Cre/Cre*^; U, U0126-treated C57BL/6J. **: *P*<0.01, *: *P*<0.05, NS: *P*>0.05.

We next explored how ERK1/2 macrophages would respond to M-CSF, which is known to trigger ERK phosphorylation among its multiple downstream signaling mechanisms. We compared results from ERK1/2 macrophages to control macrophages with intact ERK1 and ERK2 expression (from wild-type C57BL/6J or LysM mice), macrophages with single knockout of ERK1 or ERK2, or macrophages incubated with the highly specific MEK inhibitor U0126 [25] to comprehensively inhibit ERK signaling. We observed that M-CSF triggered rapid phosphorylation of ERK (peak at 5–15 min, Fig 2A). Densitometric analyses revealed that the ratios of p-ERK2 to ERK2 were 0.72 for LysM macrophages, 1.52 for E1 macrophages and 2.05 for ERK1/2 macrophages. However, due to the reduced levels of total ERK2, ERK1/2 macrophages had decreased total phosphorylated ERK levels relative to wild-type or single knockout macrophages (Fig 2B); as expected, U0126 fully blocked ERK phosphorylation. We studied the induction of two genes that are known to be induced by M-CSF and regulated by ERK, *Cd33* [22] and *Dusp5* [26]. Induction of these genes by M-CSF was reduced in ERK1/2 macrophages relative to LysM macrophages and in wild-type macrophages by treatment with U0126 (Fig 2C and 2D). M-CSF receptor was similarly expressed under all these conditions (Fig 2E), indicating that the explanation for altered gene induction by M-CSF was due to altered intracellular signal transduction as a consequence of reduced ERK expression rather than altered M-CSF receptor expression. Thus, ERK signaling is required for M-CSF induction of some genes.

### Growth of macrophages is reduced in M-CSF stimulated cultures of ERK1/2 bone marrow progenitors

**Fig 3:**
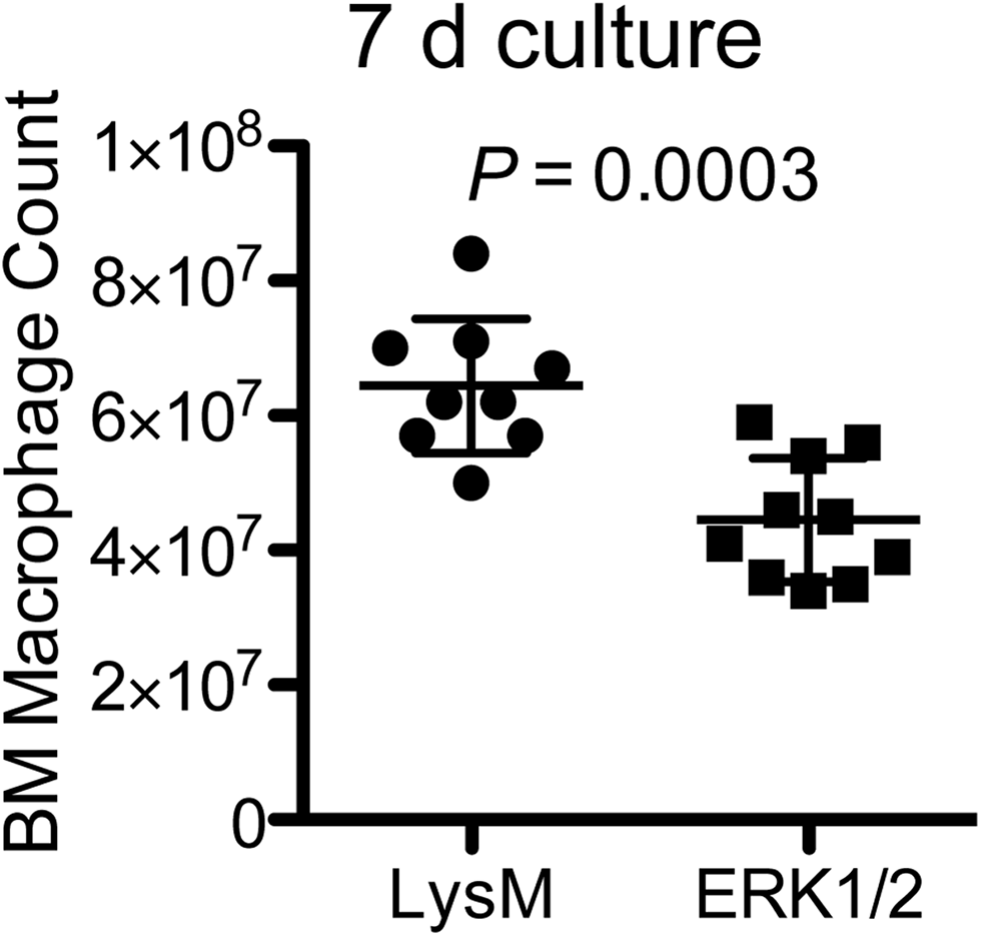
Macrophage yield from M-CSF-stimulated bone marrow cultures is reduced in ERK1/2 mice. 2.5×10^7^ bone marrow cells from ten individual mice of each genotype (LysM and ERK1/2) were cultured for 7 d in 50 ml D10F with 25% LADMAC cell conditioned medium, and macrophage yields were determined by cell count with Trypan Blue exclusion.

Since M-CSF-driven ERK signaling and gene induction were reduced in ERK1/2 macrophages, we hypothesized that the ERK-dependent growth and differentiation signals produced by M-CSF would be reduced in myeloid precursors with lowered ERK expression. Accordingly, we performed quantitative assessment of macrophage yield from bone marrow of ERK1/2 mice and control LysM mice cultured with M-CSF-containing LADMAC-conditioned medium to drive macrophage progenitor proliferation and differentiation. Cultures were initiated with 2.5×10^7^ bone marrow cells; the mean yield was 6.4×10^7^ macrophages from LysM bone marrow versus 4.5×10^7^ macrophages for ERK1/2 bone marrow (Fig 3). These data, coupled with the finding that ERK1/2 macrophages from these cultures retain expression of ERK2 (Fig 1), suggest that M-CSF-driven macrophage precursor proliferation, survival or macrophage differentiation depends on ERK expression and function.

### Macrophages from LysM and ERK1/2 mice have normal expression of characteristic macrophage markers and normal functions in response to inflammatory stimuli

**Fig 4:**
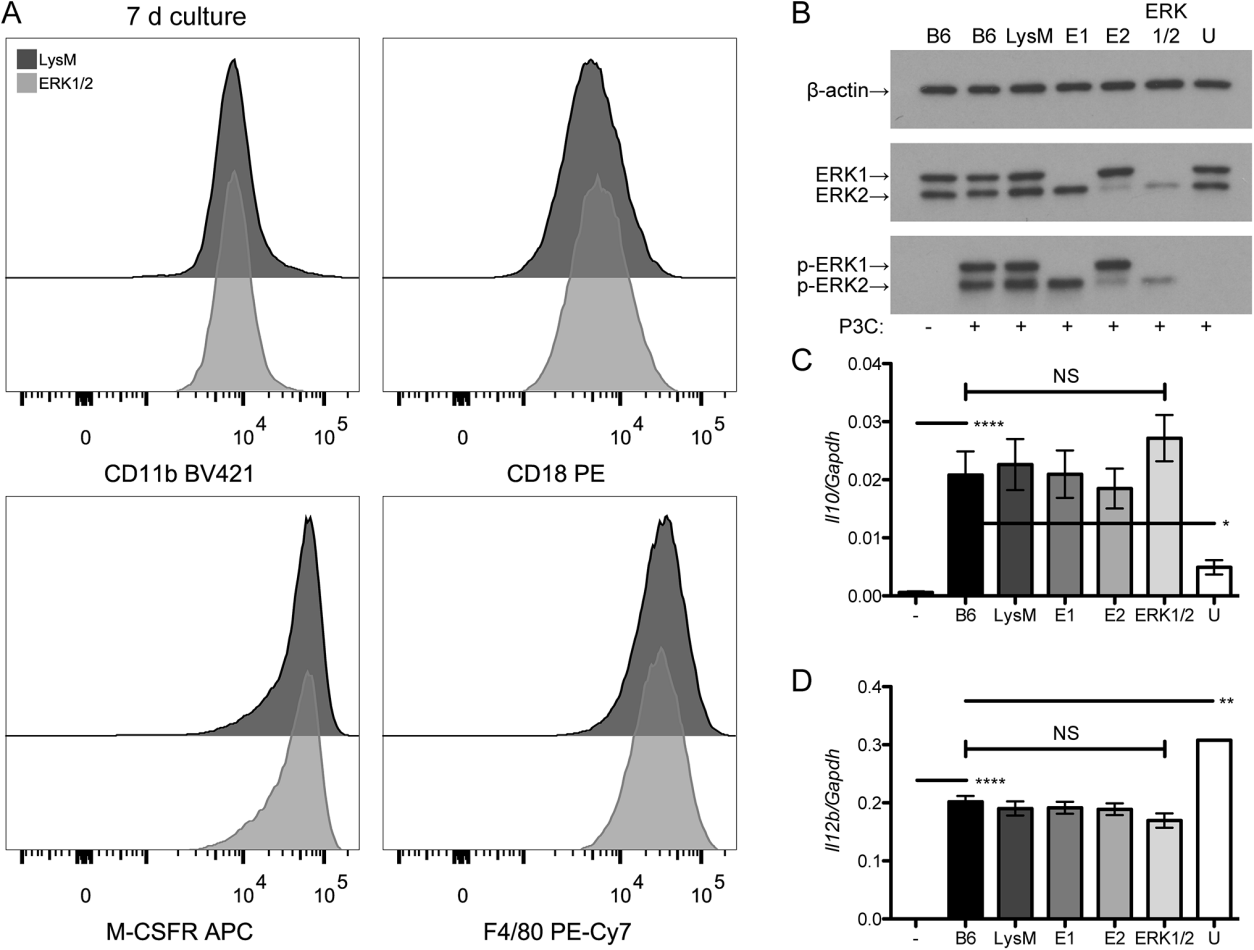
Macrophage marker expression and responses to TLR2 stimulation are intact in ERK1/2 macrophages. (A) Bone marrow-derived macrophages grown in LADMAC medium for 7 d were rested overnight in D10F, and then stained for CD11b, CD18, M-CSF receptor and F4/80, and analyzed by flow cytometry. Data are representative of two independent experiments. (B) Bone marrow-derived macrophages from LADMAC-stimulated cultures were rested overnight in D10F, pre-treated for 30 min with U0126 or DMSO vehicle (all others), and then treated with or without 30 nM Pam_3_CSK_4_ for 15 min, lysed, and analyzed for ERK phosphorylation by Western blotting. Densitometric values (in arbitrary units) for LysM, E1 and ERK1/2, respectively, were 33.12, 30.29 and 36.6 for β-actin; 35.2, 26.5 and 2.94 for ERK2; and 28.88, 34.69 and 6.36 for p-ERK2. Results are representative of two independent experiments. (C) and (D). Bone marrow-derived macrophages were pre-treated with U0126 or DMSO as in (B) and then treated with 30 nM Pam_3_CSK_4_ for 2 h (C) or 4 h (D). Gene expression was measured by qRT-PCR. These time points were previously determined to demonstrate maximal expression of *Il10* and *Il12b*, respectively [21]. Results represent two independent experiments and show means ± standard deviations. Labels: -, untreated; +, treated with Pam_3_CSK_4_; B6, C57BL/6J; E1, *Erk1* ^*-/-*^; E2, *Erk2* ^*flox/flox*^ *Lyz2* ^*Cre/Cre*^; U, U0126-treated C57BL/6J. ****: *P*<0.0001, **: *P*<0.01, *: *P*<0.05, NS: *P*>0.05.

Given the reduced yield of bone marrow-derived macrophages from ERK1/2 mice, we sought to assess whether ERK1/2 and control macrophages differed in their expression of macrophage markers or their responses to stimulation of TLR2, an innate immune receptor that triggers ERK-dependent effects among its downstream signaling pathways [27, 28]. We did not observe any difference between ERK1/2 and control macrophages in expression of CD11b, CD18, M-CSF receptor, or F4/80 antigen in macrophages (Fig 4A). We have previously studied the role of the ERK pathway in TLR2 signaling to regulate *Il10* and *Il12b* in macrophages; ERK signaling induces *Il10* expression and suppresses *Il12b* expression [21]. Stimulation with the TLR2 agonist lipopeptide, Pam_3_CSK_4_, induced ERK phosphorylation in ERK1/2 macrophages; only U0126-treated macrophages showed a lack of ERK phosphorylation. The ratio of p-ERK2 to ERK2 was 0.82 for LysM macrophages, 1.31 for E1 macrophages and 2.16 for ERK1/2 macrophages. Thus, in the absence of ERK1, there is a compensatory increase in the ratio of p-ERK2 to ERK2, and in the ERK1/2 condition, where there is very little ERK2 expression the ratio of p-ERK2 to ERK2 is greatly increased. Despite the variation in ERK phosphorylation, ERK1/2, wild-type and single knockout macrophages all showed similar induction of *Il10* and *Il12b* in response to TLR2 stimulation (Fig 4C and 4D). As predicted by our prior studies, U0126-treated macrophages showed reduced induction of *Il10* and enhanced induction of *Il12b* under ERK control (Fig 4C and 4D). We conclude that the retained amount of ERK2 in ERK1/2 macrophages is sufficient to become phosphorylated and transmit a signal to affect gene expression in response to TLR2 stimulation, allowing normal ERK-dependent regulation of *Il10* and *Il12b* downstream of TLR2. These findings suggest that ERK1/2 macrophages, although produced in lower numbers than wild-type macrophages, are phenotypically similar to wild-type macrophages in these parameters, likely due to retained expression and functionality of ERK2.

### Granulocyte-macrophage progenitors are increased in ERK1/2 mice

**Fig 5:**
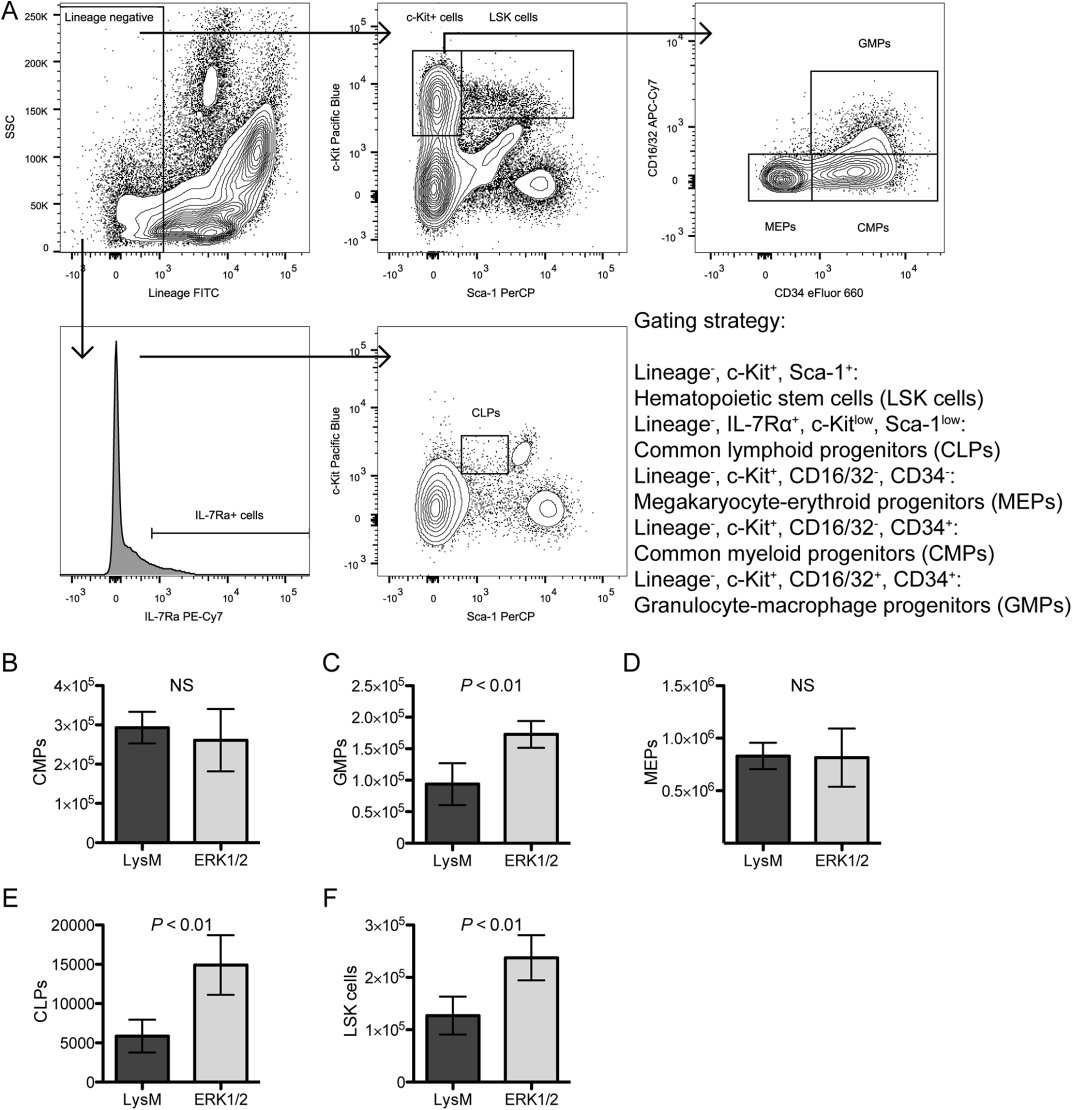
ERK1/2 mice have increased numbers of hematopoietic stem cells (LSK cells), common lymphoid progenitors, and granulocyte-macrophage progenitors on *ex vivo* flow cytometry analysis of progenitor populations. (A) Bone marrow cells from LysM and ERK1/2 mice (six of each) were counted using a hemacytometer and then analyzed by flow cytometry using the indicated gating strategy and the antibody panel described in Table 1. (B-F) Cell numbers from each population of immature hematopoietic cells were extrapolated from the total bone marrow cell counts and the percentage of each population following analysis in FlowJo. Data are represented as means ± standard deviations. Results are representative of two independent experiments.

We next explored whether populations of lineage progenitors were altered in ERK1/2 mice by using flow cytometry to enumerate bone marrow progenitor cells. The gating strategy is shown in Fig 5A (live cells were identified based on FSC/SSC and negative staining with the LIVE/DEAD Yellow reagent). Cell enumerations were determined by multiplying the percentages of cells found in the gating analysis by the total number of bone marrow cells analyzed for each individual mouse (six of each genotype, LysM and ERK1/2). We found significant increases in granulocyte-macrophage progenitors (GMPs) (Lin^-^, c-Kit^+^, Sca–1^-^, CD34^+^, CD16/32^+^, Fig 5C), common lymphoid progenitors (CLPs) (Lin^-^, IL-7Rα^+^, Sca-1^low^, c-Kit^low^, Fig 5E), and hematopoietic stem cells (LSK cells, Lin^-^, c-Kit^+^, Sca–1^+^, Fig 5F). We did not observe differences in common myeloid progenitors (CMPs) (Lin^-^, c-Kit^+^, Sca–1^-^, CD34^+^, CD16/32^-^, Fig 5B) or megakaryocyte-erythroid progenitors (MEPs) (Lin^-^, c-Kit^+^, Sca–1^-^, CD34^-^, CD16/32^-^, Fig 5D). CLPs are an early branch point in hematopoiesis and give rise to all mature lymphocytes but not to any myeloid cells [29], while MEPs are a later branch point after the commitment decision of myeloid cells to megakaryocyte or erythroid differentiation versus myeloid white blood cell differentiation [30]; neither of these populations should be ERK2 deficient due to their lineage commitment and predicted lack of LysM-Cre expression [13], and the increase in CLPs may reflect the global ERK1 deficiency affecting those cells. As GMPs represent a more committed population derived from CMPs, and GMPs give rise to the mature monocyte-macrophage and granulocyte lineages [30], the significant increase observed in GMPs implies that the macrophage growth defect in ERK1/2 mice is not due to reductions in monocyte-macrophage progenitors. We observed an increase in LSK cells in ERK1/2 mice, indicating that there is not a global hematopoietic defect that could possibly explain reductions in mature macrophages.

**Table 1:** Antibodies used for flow cytometry.

Antigen (alternate name)	Clone Name (isotype)	Fluorochrome conjugate	Vendor	Experimental Panel^a^
B220 (CD45R)	RA3-6B2 (rat IgG2a, κ)	PE-Cy7	Biolegend	Lineage, FACS
CD3ε	145-2C11 (Armenian hamster IgG1, κ)	PE-CF594	BD Biosciences	Lineage
CD4	RM4-5 (rat IgG2a, κ)	FITC	Biolegend	Lineage
CD8α	53–6.7 (rat IgG2a, κ)	APC-H7	BD Biosciences	Lineage
CD11b	M1/70 (rat IgG2b, κ)	BV421, Alexa Fluor 647, FITC, PE-Cy7	Biolegend	Lineage, BMM, FACS, Differentiation
CD16/32	93 (rat IgG2a, λ)	APC-Cy7	Biolegend	Progenitors
CD18	M18/2 (rat IgG2a, κ)	PE	Biolegend	BMM
CD34	RAM34 (rat IgG2a, κ)	eFluor 660	eBioscience	Progenitors
CD115 (M-CSFR)	AFS98 (rat IgG2a, κ)	APC	Biolegend	BMM
CD117 (c-Kit)	2B8 (rat IgG2b, κ)	Pacific Blue	Biolegend	Progenitors
CD127 (IL-7Rα)	A7R34 (rat IgG2a, κ)	PE-Cy7	Biolegend	Progenitors
CD138	281–2 (rat IgG2a, κ)	BV421	Biolegend	Lineage
F4/80	BM8 (rat IgG2a, κ)	PE-Cy7	Biolegend	BMM
Gr–1 (Ly-6G/Ly-6C)	RB6-8C5 (rat IgG2b, κ)	PerCP-Cy5.5, APC	Biolegend	Lineage, FACS, Differentiation
Lineage cocktail	See footnote^b^	FITC	Biolegend	Progenitors
Sca–1 (Ly-6A/E)	D7 (rat IgG2a, κ)	PerCP	Biolegend	Progenitors
TER–119	Ter–119 (rat IgG2b, κ)	BV510, PE-Cy7	Biolegend	Lineage, FACS
Fc Block (CD16/32)	2.4G2 (rat IgG2b, κ)	Unconjugated	BD Biosciences	BMM

^a^Experimental panels refer to: Lineage, bone marrow lineage committed cell populations; FACS, bone marrow myeloid cell sorting by FACS; BMM, cultured bone marrow-derived macrophage immunophenotyping; Differentiation, *in vitro* myeloid lineage differentiation from Lin^-^ cells; Progenitors, bone marrow hematopoietic progenitor populations.

^b^Lineage cocktail consists of a mixture of FITC-conjugated anti-CD3ε (clone 145-2C11), anti-Gr–1 (clone RB6-8C5), anti-CD11b (clone M1/70), anti-B220 (clone RA3-6B2), and anti-TER–199 (clone Ter–119) and was used according to the manufacturer’s instructions.

### Generation of monocytes and granulocytes persists in ERK1/2 bone marrow with retention of ERK2 specifically in monocytes

**Fig 6:**
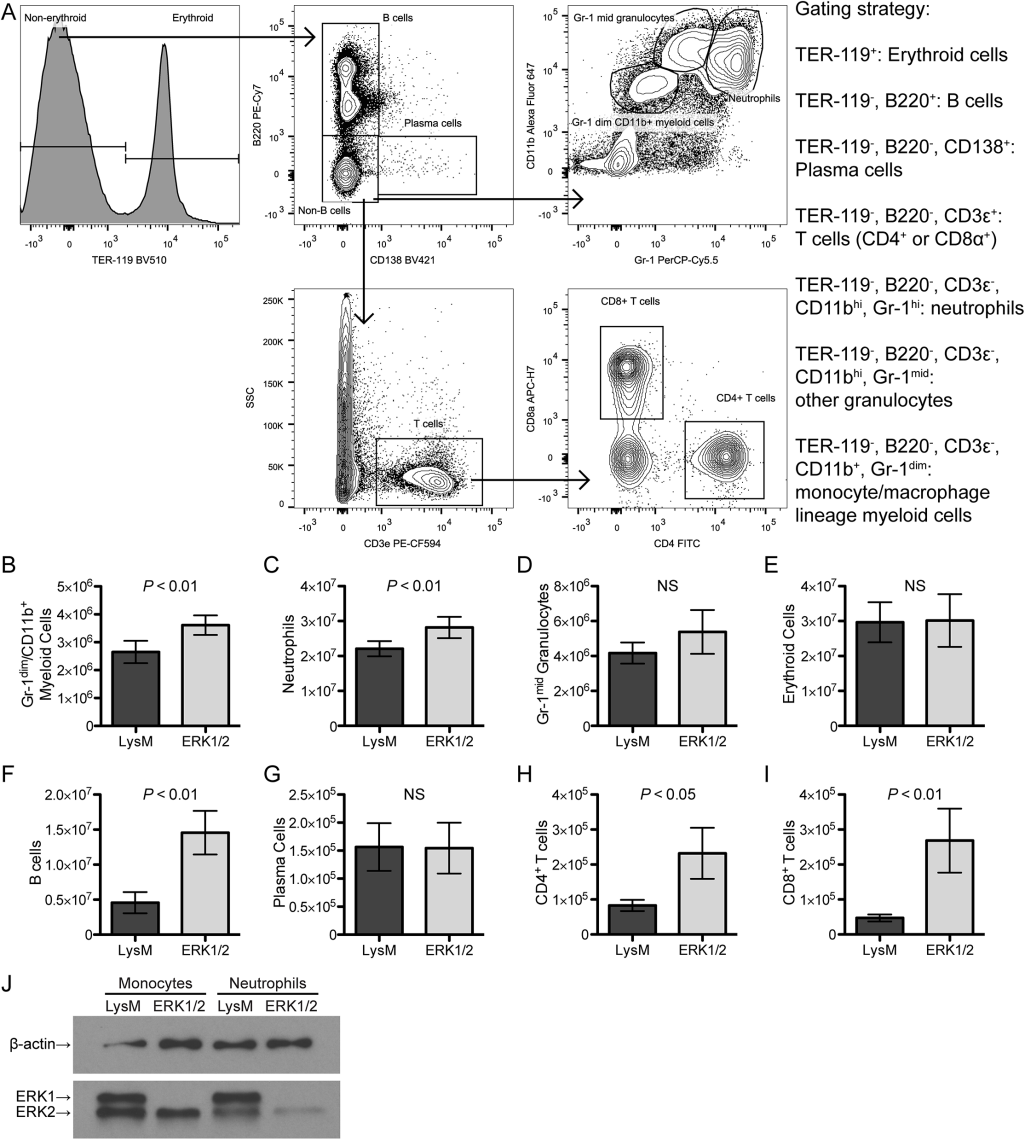
Bone marrow of ERK1/2 mice contains increased numbers of mature myeloid and lymphoid cells with retention of ERK2 expression in monocytes but not granulocytes. (A) Bone marrow cells from LysM and ERK1/2 mice (six of each) were analyzed by *ex vivo* flow cytometry to assess lineage-committed cells. (B-I) Cell numbers were calculated from total bone marrow cell counts and the percentage of each population in the gate. (J) Neutrophils (CD11b^hi^ Gr-1^hi^) and monocytes (CD11b^+^ Gr–1^-^) from LysM and ERK1/2 bone marrow were collected by FACS and analyzed by Western blotting for expression of ERK and β-actin. Data are expressed as means ± standard deviations. Results are representative of two independent experiments.

We next explored whether there was any defect in production of mature hematopoietic cells in the bone marrow of ERK1/2 mice. The gating strategy is shown in Fig 6A, and the analysis approach was as described for Fig 5. Monocyte-macrophage lineage committed cells were increased in number in the bone marrow of ERK1/2 mice as compared to LysM mice (Fig 6B). These cells were defined by intermediate expression of CD11b (a myeloid lineage marker) and low expression of Gr–1 (a granulocyte marker). We also observed an increase in neutrophil granulocytes (high expression of both Gr–1 and CD11b) in ERK1/2 mice (Fig 6C). Other Gr–1 positive cells (representing other types of myeloid cells, such as eosinophils, basophils, or immature neutrophils, Fig 6D) and erythroid cells (Fig 6E) were not altered in number in ERK1/2 mice. B lymphocytes (B220 positive) and T lymphocytes (CD3ε positive and either CD4 or CD8α single positive, reflecting mature circulating T cells) were significantly increased in ERK1/2 mice (Fig 6F, 6H and 6I); this may reflect the effects of global ERK1 deficiency in these mice on lymphocytes, since ERK2 expression should be unaffected in the lymphoid lineage, and is also reflective of increased CLPs (Fig 5E). Finally, plasma cells were not altered in number in ERK1/2 mice (Fig 6G).

FACS-purified bone marrow neutrophils (CD11b^hi^ Gr-1^hi^) and monocytes (CD11b^+^ Gr–1^-^) were analyzed for expression of ERK1 and ERK2 by Western blotting. ERK1/2 monocytes expressed wild-type levels of ERK2 protein, while ERK1/2 neutrophils had greatly reduced ERK2 expression (Fig 6J). The numbers of neutrophils and monocytes were increased in the bone marrow of ERK1/2 mice (Fig 6B and 6C). The retention of ERK2 expression in monocytes suggests that either Cre recombinase activity is expressed only after this stage of maturation (e.g. in macrophages that exhibit selective growth or survival of a subset retaining ERK2 expression; a likely explanation, see Discussion) or, if Cre recombinase expression has occurred by this stage, there is selective survival/growth of a subset of monocytes that have retained ERK2 expression. Together, these data support the conclusion that ERK activity is dispensable for the generation of neutrophils but required for generation of macrophages.

### *In vitro* culture of ERK1/2 hematopoietic progenitors produces mature granulocytes and monocytes/macrophages at slightly reduced levels

**Fig 7:**
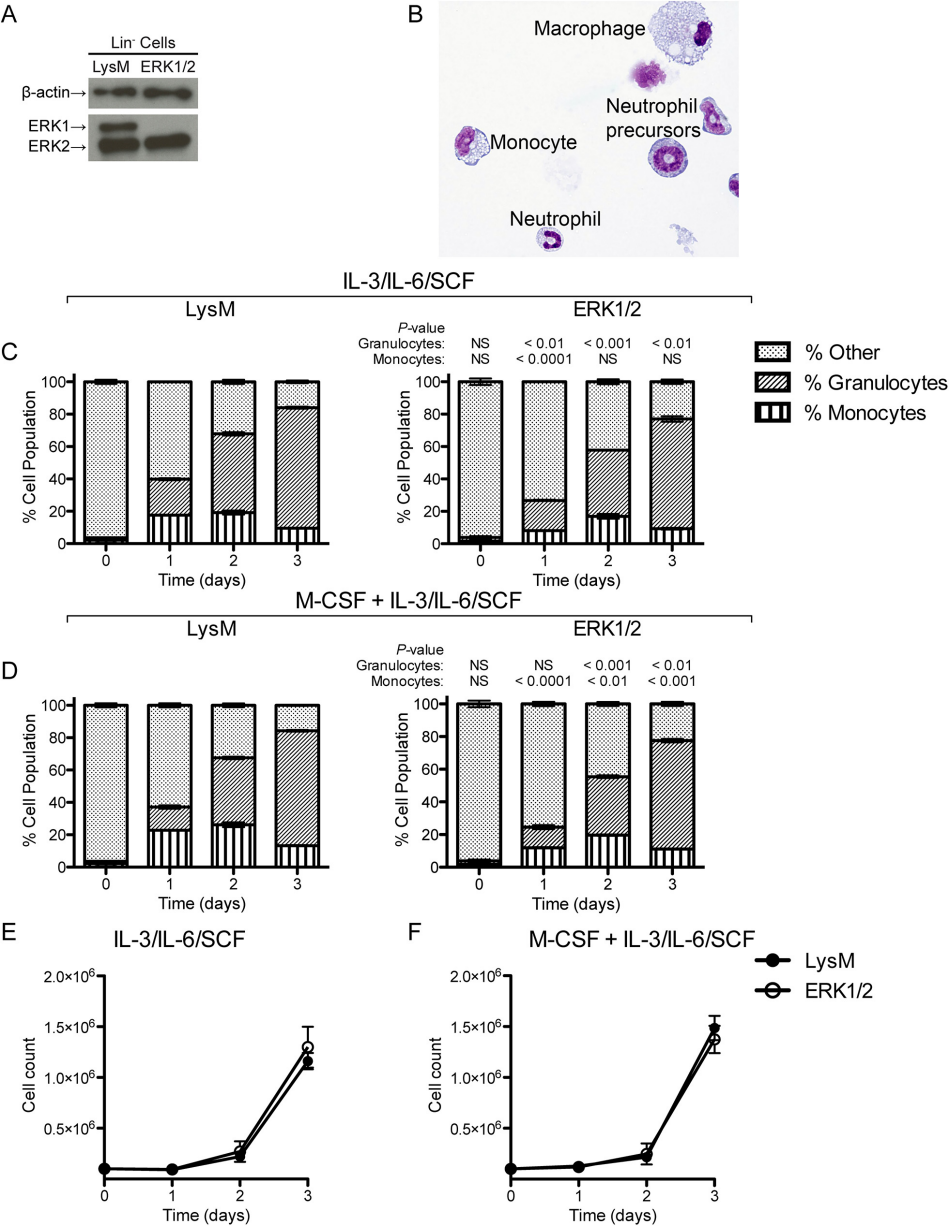
Production of monocytes and macrophages by *in vitro* culture of Lin^-^ progenitors from ERK1/2 and LysM bone marrow. (A) Western blot of Lin^-^ cells from LysM and ERK1/2 bone marrow. (B) Cytospin preparation showing representative cells generated by culture of Lin^-^ cells for 4 d in 5 ng/ml IL–3, 10 ng/ml IL–6, and 10 ng/ml SCF (similar results were observed with M-CSF in addition to these growth factors). (C) Lin^-^ cells were grown in IL–3, IL–6, and SCF and monitored daily for acquisition of neutrophil (CD11b^+^ Gr–1^+^) and monocyte (CD11b^+^ Gr–1^-^) lineage markers by flow cytometry. (D) Cells were grown and analyzed as in (C) but with the addition of 5 ng/ml M-CSF. (E) and (F) Total yields of cells from cultures of Lin^-^ cells in myeloid-stimulating cytokines. Data in (A) and (B) are representative of two independent experiments; data in (C)-(F) show the means ± standard deviations of triplicate samples and are representative of two independent experiments.

We sought to explore whether *in vitro* culture of Lin^-^ ERK1/2 bone marrow progenitors under conditions that included both M-CSF and other growth factors would produce granulocytes and monocytes. We isolated Lin^-^ cells, representing hematopoietic stem cells and lineage-committed progenitors, from LysM and ERK1/2 bone marrow. As predicted, ERK2 expression was intact in these cells (Fig 7A; LysM-driven Cre expression and deletion of ERK2 are not predicted at this stage of differentiation). Cultures of these cells in IL–3, IL–6 and SCF, with or without M-CSF, produced neutrophil precursors, mature neutrophils, monocytes, and macrophages (Fig 7B). Flow cytometry analysis of IL–3/IL–6/SCF-stimulated cultures revealed slightly reduced generation of granulocytes from ERK1/2 progenitors (relative to control LysM progenitors) that persisted through day 3 and a transient slight reduction of monocytes from ERK1/2 progenitors (manifested at day 1 only) (Fig 7C). With the addition of M-CSF, generation of both monocytes and granulocytes was slightly diminished in ERK1/2 cultures relative to LysM cultures (Fig 7D). However, both monocytes and granulocytes were generated from both LysM and ERK1/2 progenitors under all of these conditions, and the total cell numbers were not significantly altered by these conditions (Fig 7E and 7F). Thus, Lin^-^ progenitors from ERK1/2 bone marrow have the potential to generate mature granulocytes and monocytes when stimulated with other growth factors, although the full production of monocytes/macrophages may require selective retention of ERK2 expression when M-CSF is a primary driver, as demonstrated in earlier figures.

### An increase in neutrophils is the only notable change in peripheral blood cell population numbers in ERK1/2 mice

Finally, we assessed potential deficits in peripheral blood cell populations by performing complete blood counts on ERK1/2 mice and LysM control mice. ERK1/2 mice showed slightly elevated neutrophil counts (1.5×10^3^/μL for ERK1/2 mice versus 1.1×10^3^/μL for LysM mice, Table 2). Otherwise, ERK1/2 and LysM mice showed equivalent numbers of total leukocytes, lymphocytes, monocytes, platelets, and erythrocytes, as well as similar erythrocyte indices (Table 2). ERK1/2 mice showed a very slightly reduced MCV and concomitant increase in MCHC, not thought to be biologically relevant. These data indicate neither profound defects in development along the major hematopoietic lineages nor defects in egress of mature cells from the bone marrow. An increase in neutrophil number has been reported in the setting of decreased tissue macrophage numbers or functional suppression of macrophages [31]. The peripheral blood monocyte count was normal in ERK1/2 mice compared to LysM controls, and so the neutrophilia observed in these mice may relate more to altered tissue macrophage numbers or function.

**Table 2:** Complete blood count data.

	LysM, N = 7	ERK1/2, N = 8	*P*
Total WBC, ×10^3^/μL	8.7 ± 2.0	9.1 ± 2.1	NS
Neutrophils, ×10^3^/μL	1.1 ± 0.32	1.5 ± 0.37	<0.05
Lymphocytes, ×10^3^/μL	7.3 ± 1.7	7.1 ± 1.7	NS
Monocytes, per μL	346 ± 98	344 ± 69	NS
Total RBC, ×10^6^/μL	9.9 ± 1.4	10.3 ± 0.83	NS
Hemoglobin, g/dL	13.5 ± 2.0	14.1 ± 1.1	NS
Hematocrit, %	44.9 ± 7.0	45.3 ± 3.8	NS
MCV, fL	45.2 ± 0.78	43.8 ± 0.96	<0.01
MCH, pg	13.6 ± 0.20	13.6 ± 0.39	NS
MCHC, g/dL	30.2 ± 0.59	31.2 ± 0.75	<0.05
RDW, %	17.5 ± 0.46	17.9 ± 0.48	NS
Platelets, ×10^3^/μL	660 ± 110	674 ± 250	NS
MPV, fL	4.5 ± 0.22	4.7 ± 0.34	NS

Data are displayed as means ± standard deviations. NS indicates not significant, *P*>0.05. Abbreviations: WBC, white blood cells; RBC, red blood cells; MCV, mean corpuscular volume; MCH, mean corpuscular hemoglobin; MCHC, mean corpuscular hemoglobin concentration; RDW, red blood cell distribution width; MPV, mean platelet volume.

Together, these experiments did not reveal any defects in hematopoietic lineages in ERK1/2 bone marrow progenitors, did not identify any reductions in mature bone marrow cells, and did not detect any defects in the peripheral blood. We instead found increased numbers of GMPs and their direct differentiation products, neutrophils and monocyte-macrophage lineage myeloid cells, and a concomitant increase in circulating neutrophils in ERK1/2 mice. The lack of evidence for a defect in myeloid progenitors, or growth arrest along the myeloid maturation pathway, indicates instead a cell-intrinsic signaling defect due to reduced ERK expression in ERK1/2 monocytes and macrophages that affects cell proliferation and development late in the macrophage development pathway.

## Discussion

In this paper, we describe a novel genetic approach that demonstrates a requirement for ERK signaling for monocyte/macrophage development. Prior studies have reported that ERK, PI3K/Akt and other effector kinases participate in signaling in response to M-CSF [8, 10], but the exact contributions and potential required roles of the ERK pathway for development of macrophages have remained undetermined. Prior studies were also limited to modeling M-CSF signaling with cell lines, or were more focused on the requirements of specific tyrosine phosphorylation sites of the M-CSF receptor. This study expands knowledge about the role of the ERK pathway in macrophage development using a novel mouse model and reveals a critical role for ERK in development of primary macrophages.

We produced *Erk1* ^*-/-*^ *Erk2* ^*flox/flox*^ *Lyz2* ^*Cre/Cre*^ mice that were hypothesized to lack both ERK1 and ERK2 isoforms in the myeloid lineage, based on prior reports of expression pattern of lysozyme M and use of *Lyz2* ^*Cre/Cre*^ to delete floxed genes in macrophages. Surprisingly, ERK1/2 macrophages expressed ERK2, despite the fact that *Erk2* ^*flox/flox*^ *Lyz2* ^*Cre/Cre*^ macrophages, which expressed ERK1, showed efficient deletion of ERK2 (Fig 1B). These findings indicate a selective pressure to retain ERK2 expression for development of macrophages when ERK1 is not present and imply a requirement for ERK1 or ERK2 for macrophage development. This interpretation suggests that precursor cells lacking both ERK1 and ERK2 do not survive or differentiate into macrophages, limiting macrophage production to selective outgrowth of a small proportion of progenitors that had failed to delete floxed *Erk2*. *Erk2* ^*flox/flox*^ *Lyz2* ^*Cre/Cre*^ macrophages lacked ERK2 expression, showing that *Lyz2*-driven Cre recombinase can delete the *Erk2* gene and suggesting that continued ERK1 expression facilitates macrophage growth and survival under these circumstances (Fig 1B). We hypothesize that macrophages that develop from ERK1/2 bone marrow derive from precursors with incomplete Cre-mediated *Erk2* deletion, which may represent deletion of one allele and not both. We also hypothesize that precursor cells that are completely ERK1 and ERK2 null, if produced, cannot grow efficiently or differentiate into macrophages due to inability of M-CSF to transmit critical growth signals through ERK.

We considered the possibility that ERK was required to maintain normal numbers of bone marrow progenitors. This possibility was unlikely, as *Lyz2* ^*Cre/Cre*^ does not operate early in hematopoiesis, so progenitors should express ERK2 until late in myeloid fate commitment. Consistent with this hypothesis, ERK1/2 monocytes expressed wild-type levels of ERK2 protein (Fig 6J). We found no reductions in progenitors or mature lineage cells in the bone marrow of ERK1/2 mutant mice (Figs 5 and 6). In fact, we observed that the bone marrow in ERK1/2 mice had increased numbers of immature (Fig 5C) and mature myeloid cells (Fig 6B and 6C), as well as increased numbers of LSK cells (Fig 5F) relative to bone marrow in LysM control mice. We also observed normal levels of circulating monocytes in ERK1/2 mice (Table 2). These data indicate that the reduced production of macrophages reflects a defect in cell proliferation and differentiation in the terminal stages of macrophage development that are driven by M-CSF and require ERK expression.

ERK1/2 macrophages were phenotypically and functionally equivalent to wild-type macrophages in some of the assays we employed. We examined expression of mature macrophage markers and found no differences (Fig 4A). We studied cytokine responses to the innate immune stimulus Pam_3_CSK_4_, which activates TLR2, and found no differences in regulation of *Il10* or *Il12b* expression, two cytokine transcripts regulated by ERK (Fig 4B and 4D). We conclude that ERK1/2 macrophages, once produced, are phenotypically and functionally similar to wild-type macrophages in these parameters. We observed a compensatory increase in the proportion of ERK2 that becomes phosphorylated in ERK1/2 cells (which retain only a low level of ERK2 expression) (Fig 4B); apparently the low level of p-ERK achieved in these cells is sufficient to allow normal regulation of *Il10* or *Il12b* expression.

On the other hand, investigation of ERK signaling in response to M-CSF revealed defects in ERK activation and ERK-regulated gene induction in ERK1/2 macrophages. As compared to wild-type macrophages, which had full ERK1 and ERK2 expression, ERK1/2 macrophages, which had very limited ERK2 expression, displayed reduced ERK2 phosphorylation (although a greater proportion of the remaining ERK was phosphorylated) (Fig 2B) and reduced expression of *Cd33* and *Dusp5* (Fig 2C and 2D), two genes regulated by ERK downstream of M-CSF. Finally, we did not observe differences in expression of M-CSF receptors, which could theoretically reduce M-CSF responsiveness, either at baseline (Fig 4A) or following 24 h of M-CSF stimulation (Fig 2E). These results together point towards an intrinsic signal transduction defect most likely due to reduced but not eliminated ERK2 protein in ERK1/2 macrophages.

In summary, we report a novel approach for studying the ERK signaling pathway in macrophages, namely *Erk1* ^*-/-*^ *Erk2* ^*flox/flox*^ *Lyz2* ^*Cre/Cre*^ triple mutant mice. We found that ERK2 expression was reduced but not fully eliminated in macrophages from these mice, and that macrophages from these mice exhibited an overall growth defect under M-CSF-driven conditions. We also found that M-CSF signaling through the ERK pathway was deficient in macrophages from these mice. Thus, the M-CSF drive for macrophage proliferation requires ERK. These results give the first indication that the ERK signaling pathway is essential for M-CSF-driven macrophage production. These findings may also contribute to our understanding of the development or growth requirements of monocytic forms of myeloid malignancies and may lead to new therapeutic targets, since ERK signaling is known to contribute to cancer growth, and inhibitors of members of the MAPK cascade are in development for solid tumors [32, 33].

## Supporting Information

S1 ARRIVE Checklist ARRIVE checklist for this study. (PDF)
